# Adenoid Cystic Carcinoma of the Breast: A Narrative Review and Update on Management

**DOI:** 10.3390/cancers17071079

**Published:** 2025-03-23

**Authors:** Taylor Neilson, Zaibo Li, Christina Minami, Sara P. Myers

**Affiliations:** 1 Division of Surgical Oncology, Department of Surgery, The Ohio State University, Columbus, OH 43210, USA; taylor.neilson@osumc.edu; 2 Department of Pathology, The Ohio State University Wexner Medical Center, Columbus, OH 43210, USA; zaibo.li@osumc.edu; 3 Division of Breast Surgery, Department of Surgery, Brigham and Women’s Hospital, Boston, MA 02115, USA; cminami@mgb.org; 4 Breast Oncology Program, Dana-Farber Brigham Cancer Center, Boston, MA 02215, USA; 5 Harvard Medical School of Medicine, Boston, MA 02115, USA; 6 Comprehensive Cancer Center, The Ohio State University, Columbus, OH 43210, USA; 7 CATALYST, Center for the Advancement of Team Science, Analytics, and Systems Thinking in Health Services and Implementation Science Research, College of Medicine, The Ohio State University, Columbus, OH 43210, USA

**Keywords:** breast cancer, adenoid cystic carcinoma

## Abstract

Rare breast malignancies represent a challenge for treatment decision making given the lack of evidence-based guidelines. As a particularly uncommon tumor, adenoid cystic carcinomas are especially challenging. Although, histopathologically, they share the same tumor molecular profile as hormone receptor-negative and HER2 nonamplified carcinomas with aggressive physiology, adenoid cystic carcinomas generally have a favorable prognosis. Thus, there is evidence to suggest that more aggressive treatment regimens may not provide better therapeutic effects. In this review, we discuss ACCB with the goal of highlighting pathophysiology, clinical features, and treatment strategies. Existing data support consideration for adjuvant radiation with the omission of axillary staging and systemic therapies.

## Simple Summary

Adenoid cystic carcinoma rarely occurs in the breast. Despite having a favorable prognosis, these carcinomas share a tumor molecular profile that is similar to aggressive subtypes of commonly occurring breast cancers. The majority of these aggressive subtypes require multimodal treatment involving chemotherapy. This review of the literature describes the pathophysiology, clinical features, and treatment of adenoid cystic carcinoma of the breast, highlighting opportunities for the de-escalation of management.

## 1. Introduction

Breast cancer, the second most common malignancy among US women, is a leading cause of cancer-related mortality [1]. The majority of breast cancers originate from the primary anatomical areas of the breast; ductal carcinomas represent 70–80% of breast cancers, while lobular carcinomas account for 5–15%. Because less-common histopathologic subtypes are responsible for only 2% of tumors in the breast, their management can be challenging given the lack of evidence-based guidelines to assist with multidisciplinary management [2].

A particularly uncommon tumor, adenoid cystic carcinoma of the breast (ACCB), accounts for <0.1% of all breast malignancies [3]. Given its rarity, treatment protocols are often extrapolated from case studies and small series, emphasizing the need for more comprehensive studies and evidence-based guidelines. In this review, we discuss ACCB with the goal of highlighting pathophysiology, clinical features, and treatment strategies.

## 2. Epidemiology and Clinical Presentation

While increasing overall for breast cancer, the age-adjusted incidence ratio of ACCB appears stable [4]. Epidemiologically, the majority of cases are diagnosed in White women, with the peak distribution of age at diagnosis in the 6th–7th decades of life [4]. Although lesions can present as an asymmetry or irregular high-density mass without calcifications on mammography, they often lack a characteristic appearance and may be misdiagnosed as benign [5]. Ultrasound usually demonstrates a sonographic hypoechoic or heterogenous mass as a correlate for mammographic findings. MRI with gadolinium may be helpful in characterizing the tumor; Glazebrook et al. demonstrated that, in a sample of five women imaged with MRI with gadolinium, rapid and heterogenous enhancement was noted at the site of tumor in all cases [5]. T2 imaging, on the other hand, varied, with the solid variant of ACCB showing high signal intensity on these images. Positron emission tomography (PET) may be useful in distinguishing primary ACCB from metastatic disease [6].

Multifocal lesions are uncommon [3]. Approximately half of tumors occur in the subareolar location [7]. Although the majority of tumors lack estrogen/progesterone receptors (ER/PR) and HER2 amplification, unlike ductal or lobular types with triple-negative tumor molecular profile, ACCB does not exhibit an aggressive pathophysiology. Early disease at presentation is the rule rather than exception, with lymph node metastases being rare [7], and prognosis is excellent, with 10-year survival upward of 90% [8].

## 3. Histopathology

**Figure 1:**
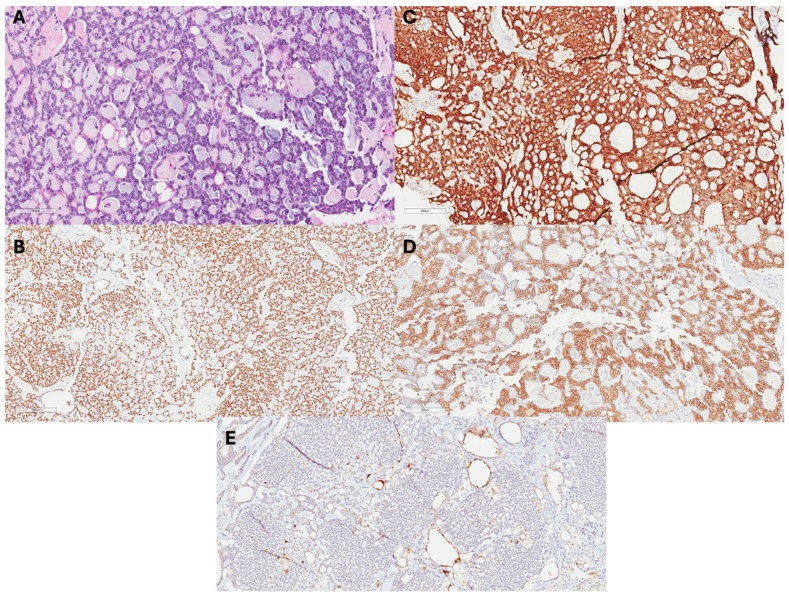
Histopathology of ACCB: (**A**) H&E images show an ACCB with a tubular growth pattern, basaloid cells, and mucoid materials in lumens. (**B**) Tumor cells + p63, (**C**) +cKit, and (**D**) +CK5 (**E**) negative for ER.

Although adenoid cystic carcinomas most commonly originate from salivary glands, they can be diagnosed in other areas of the head and neck, breast, reproductive tract, prostate, and skin [9]. Grossly, ACCB are usually well-circumscribed smaller tumors, with most being less than or equal to 5 cm in size, with pink, tan, or gray microcysts [10]. Microscopically, the staining of ACCB highlights epithelium and myoepithelium similar to that which exists in salivary adenoid cystic carcinomas (Figure 1) [10,11]. Luminal and myoepithelial–basal cells may demonstrate tubular–trabecular, cribriform, or solid–basaloid patterns that bind glandular or pseudo-glandular spaces [10]. The glandular lumina contain mucin with periodic acid-Schiff-positive staining. Additionally, immunohistochemical stains demonstrate CK7, CK8/18, epithelial membrane antigen, and CD117 positivity [5,7,8]. The pseudo-glandular structures represent invaginations of stroma and exhibit myoepithelial–basal cells that demonstrate CK5, CK5/6, CK14, and CK17 immunoreactivity, as well as p63, actin, calponin, S-100, vimentin, and epidermal growth factor receptor [7]. Furthermore, ACCB often has a lower Ki-67 index than verse adenoid cystic carcinoma from other origins, which typically has a higher Ki-67 index [12,13].

Historically, ACCB was graded similarly to salivary adenoid cystic carcinomas, with criteria reflecting the degree of solid growth. As cribriform and tubular–trabecular tumors do not have solid growth, they are considered to be grade I. Tumors with a solid–basaloid architectural pattern are considered to be grade II if they have ≤30% solid growth and grade III if they have >30% [7,14]. The most recent American Joint Committee on Cancer Staging recommendations indicate that all breast carcinomas use Nottingham histologic grading; thus, ACCB should be classified as grade 1 or 2 based on these guidelines [8].

As mentioned previously, 75% of ACCB lacks ER, PR, and HER2 amplification [15]. Unlike other breast cancers with this triple-negative tumor molecular profile (TNBC), ACCB usually represent a low-grade, low-proliferative subtype, with less than 5% of cases showing axillary involvement and fewer than 2% presenting with metastatic disease [16]. Furthermore, compared to other tumor molecular subtypes of ACCB, triple-negative disease does not appear to be a significant factor for overall survival in ACC of the breast [16]. In the largest cohort to date, Ghabach et al. observed that, among 933 patients with ACCB, overall survival at 5 years was 88% [17], with relative survival rates of 98% and 95% at 5 and 10 years, respectively. Other studies have corroborated these excellent survival rates, showing greater than 90% survival at 5 and 10 years post treatment [18,19]. Given the difference in survival compared to other TNBC cases, it has become increasingly important to develop accurate histological methods for confirming ACCB. Batra et al. recently found MYB protein overexpression in all 17 ACCB cases studied and recommend routine MYB immunohistochemistry for diagnosis [20].

## 4. Locoregional Treatment

Surgery is the mainstay of treatment for ACCB. Similarly to ductal or lobular histopathologic subtypes, breast conservation with radiation provides an acceptable alternative to mastectomy in appropriate candidates (Table 1) [16,21,22]. Radiation may have locoregional benefit after breast conservation surgery (BCS) for ACCB. In their analysis of 61 patients treated at Rare Cancer Network-affiliated centers between 1980 and 2007, Khanfir et al. characterized the 5-year locoregional control rates among 20 patients who underwent mastectomy (5 of whom received postmastectomy radiation (PMRT)) and 41 patients who underwent BCS (35 of whom received adjuvant radiation). While PMRT did not impact locoregional control after mastectomy, radiation after breast conservation was significantly associated with improved 5-year locoregional control (95% versus 83%; *p* = 0.03) [22].

In addition to locoregional control, Khanfir et al. characterized 5-year overall survival and disease-free survival for the entire patient cohort [22]. Though the surgery type was not specified, radiation was not associated with survival on univariate analysis. In two Surveillance, Epidemiology, and End Result Program (SEER)-based analyses, however, survival benefit from adjuvant radiation was observed. In their analysis of 376 patients between 1988 and 2005, Coates et al. reported that, agnostic to surgery-type, radiation was significantly associated with overall survival, with an absolute survival benefit of 9% and 21% at 5 and 10 years, respectively (*p* = 0.005) [19]. In a multivariate analysis adjusting for surgery type (BCS versus mastectomy), radiation retained significant benefit (HR 0.44, 95% CI 0.22, 0.88). When restricting the analysis to patients who had BCS, although survival differences of 12.4% and 19.7% were noted at 5 and 10 years, respectively, favoring adjuvant radiation, in the multivariable model, radiation was not significantly associated with survival. In an updated analysis of 478 patients (154 mastectomy alone, 20 mastectomy + PMRT, 107 BCS alone, and 197 BCS + adjuvant radiation) from the SEER database between 1998 and 2011, Sun et al. reported that 5-year cancer-specific survival was improved with adjuvant radiation both in cases of BCS (96.1% BCS with radiation versus 91.8% BCS alone) and mastectomy (94.1% mastectomy with radiation versus 90.2% mastectomy alone; *p*-value comparing all groups = 0.026) [23]. These studies collectively indicate that adjuvant radiation may provide a survival benefit and merits consideration with multidisciplinary discussion. More recently, in their multicenter retrospective study, Lee et al. considered locoregional recurrence patterns for 93 patients treated for ACCB with BCS (*n* = 75 (90.7%)) or mastectomy (*n* = 18 (19.3%)) [24]. In their study, 71 (94.7%) patients in the BCS group and 5 (27.8%) of patients in the mastectomy group received adjuvant radiation. At a median follow up of 50 months, seven patients experienced locoregional recurrence and twelve experienced distant disease. Of these, one case of distant disease occurred in a patient who had BCS, four locoregional and eight distant metastases occurred in patients receiving BCS with adjuvant radiation, 1 locoregional and 2 distant metastases occurred in a patient who had undergone a mastectomy alone, and two locoregional recurrences and one distant metastasis occurred in patients who received a mastectomy with adjuvant radiation. Although lymph node metastasis and poorly differentiated tumors were correlated with worse locoregional recurrence-free survival, adjuvant radiation was not.

Several investigations have discussed the surgical management of the axilla (Table 2). Khanfir et al. reported that, of the 51/61 (84%) patients in their cohort who underwent axillary management with either sentinel lymph node biopsy alone or axillary dissection, all were confirmed to be node-negative on pathology (median number of lymph nodes 8, range 1–29) [22]. As five other studies have reported a similarly low prevalence of pathologically positive lymph nodes among patients with clinically node-negative disease upon sentinel node biopsy, the omission of axillary staging for patients with ACCB may be a reasonable consideration [14,15,21,25,26].

## 5. Systemic Treatment

The role of systemic therapy for ACCB remains unclear. In their single-institution study of 28 individuals, Arpino et al. reported that 6 patients received endocrine therapy and 1 received chemotherapy with disease-free survival (DFS) at 5 years reported at 100% in patients, regardless of systemic treatment [16]. Revisiting Khanfir et al.’s data, 7/61 patients were prescribed tamoxifen and 15/61 patients received adjuvant chemotherapy (regimens included 5-fluorouracil, epirubicin, and cyclophosphamide (4 patients); cyclophosphamide, methotrexate, and 5-fluorouracil (2 patients); doxorubicin and cyclophosphamide (1 patient); and an unrecorded regimen (1 patient)) [22]. Although systemic therapies were not specifically addressed in these analyses, the 5-year overall survival rate was 94%, despite a minority having received adjuvant endocrine and/or chemotherapy. Interestingly, in their multi-institutional retrospective cohort study of 93 patients, Lee et al. reported that 39 (41.9%) patients received chemotherapy, most commonly with adjuvant doxorubicin–cyclophosphamide followed by docetaxel–cyclophosphamide. Chemotherapy was not found to be significantly associated with recurrence, locoregional recurrence-free survival, or progression-free survival [24]. Similarly, in their review of the SEER database, Li et al. did not observe an improvement in the OS (HR 1.105, *p* = 0.665) or breast cancer-specific survival (HR 3.079, OS *p* = 0.001) after adjuvant therapy administration for ACCB [26].

## 6. Conclusions

ACCB presents a unique challenge in diagnosis, treatment, and management among various breast malignancies. Despite being a form TNBC, ACCB has shown to be a less-aggressive subtype with less propensity to spread to lymph nodes and a lower metastatic potential, resulting in a generally favorable prognosis. Upfront surgical resection with or without axillary staging in patients who are clinically node-negative is indicated, with consideration for adjuvant radiation therapy. Systemic therapy may provide only a minimal benefit, without sufficient justification to merit exposure to the cytotoxic adverse effects. While further investigations are needed to define standardized treatment pathways, the rarity of ACCB serves as a major barrier. As with more common breast neoplasms, since de-escalation may challenge care coordination [29], it is imperative that the decision to omit a particular modality be reached through multidisciplinary consensus given the absence of robust data.

## Figures and Tables

**Table 1:** Summary of studies describing the role of adjuvant radiation in adenoid cystic carcinoma of the breast.

Study	N	Data Source	Treatment	Results
Coates et al. *J. Surg. Oncol.*, 2010 [19]	376	SEER 1988–2005	BCS: 60% BCS + RT: 52% Mastectomy: 40% Mastectomy + PMRT: 7%	Absolute benefit of RT: 5-year OS: 9% 10-year OS: 21% BCS 5-year OS: 12.4% BCS 10-year OS: 19.7%
Sun et al. *Breast*, 2017 [23]	478	SEER 1988–2011	BCS: 22.4% BCS + RT: 41.2% Mastectomy: 32.2% Mastectomy + PMRT: 4.2%	5-yr CSS: BCS: 91.8% BCS + RT: 96.1% Mastectomy: 90.2% Mastectomy + PMRT: 94.1%
Arpino et al. *Cancer*, 2002 [16]	28	Single institution	BCS: 4% BCS + RT: 18% Modified radical mastectomy: 64% Total mastectomy: 11% Modified radical mastectomy + RT: 4% Endocrine therapy: 21% Chemotherapy: 4%	pN+: 4% 5-year DFS: 100%
Khanfir et al. *Int J Radiat Oncol Biol Phys*, 2012 [22]	61	Rare cancer network 1980–2007	BCS: 67% RT: 66% Axillary staging: 84% Chemotherapy: 13% Endocrine therapy: 11%	pN+: 0% 5-year LRC: 95% 5-year DFS: 82% 5-year OS: 94%
Lee et al. *Cancer Res Treat*, *2025* [24]	76	Multi-institution retrospective review	BCS 80% Total mastectomy 20% BCS + RT 94.7% Total mastectomy + RT 28.8%	3-year LRFS 94% 5-year LRFS 84.2% 3-year OS 98.5% 5-year OS 94.4%

Abbreviations: SEER: Surveillance, Epidemiology, and End Result; BCS: breast conservation surgery; RT: radiation therapy; PMRT: post-mastectomy radiation therapy; OS: overall survival; CSS: cancer-specific survival; DFS: disease-free survival; pN+: pathologically node-positive; and LRC: locoregional control.

**Table 2:** Summary of studies describing axillary management in adenoid cystic carcinoma of the breast.

Study	N	Results
Arpino et al. *Cancer*, 2002 [16]	28	pN+: 1/28 patients DFS: 100% at 5 years
Kleer et al. *Am J Surg Pathol*, 1998 [27]	31	pN+: 0/20 patients DFS: 100% at 7 years
Page et al. *Cancer Res Treat*, 2005 [21]	140	pN+: 3/140 patients
Treitl et al. *Breast Cancer*, 2018 [25]	6	pN+: 0/6 DFS: 100% at 7.5 years
Millar et al. *Breast Cancer Res Treat*, 2004 [28]	9	pN+: 4 10-year LRR: 31% 10-year CSS: 10

Abbreviations: CSS: cancer-specific survival; DFS: disease-free survival; pN+: pathologically node-positive; and LRR: locoregional recurrence.
